# Pyogenic Spondylitis after Vertebroplasty - A Report of Two Cases -

**DOI:** 10.4184/asj.2007.1.2.106

**Published:** 2007-12-31

**Authors:** Chang Bum Lee, Hyung Sup Kim, Yong Jin Kim

**Affiliations:** Department of Orthopedic Surgery, Choon Hae Hospital, Busan, Korea.

**Keywords:** Vertebroplasty, Complications, Pyogenic spondylitis

## Abstract

Vertebroplasty is now extensively used worldwide for thoracic and lumbar osteoporotic compression fractures. Although percutaneous vertebroplasty is considered a minimally invasive procedure, it may result in several complications. In this report, we present two patients where pyogenic spondylitis developed after vertebroplasty surgery was required. The serious nature of these infections, surgical management and complication avoidance are discussed.

## Introduction

Galibert et al.^1^ first introduced percutaneous vertebroplasty in 1984 for the treatment of hemangiomas in the spine. Management of symptomatic osteoporotic vertebral compression fractures by vertebroplasty with the use of polymethylmethacrylate has become increasingly popular. Although vertebroplasty has been demonstrated to be a remarkably safe and effective treatment method, it is not free of complications. To the best of our knowledge, pyogenic spondylitis caused by percutaneous vertebroplasty is a rare complication. As far as we know, infectious complications requiring surgical management are particularly rare. We report here two cases of pyogenic spondylitis that occurred after the patient had undergone vertebroplasty, with an accompanying review of the literature.

## Case Reports

### Case 1

**Fig. 1:**
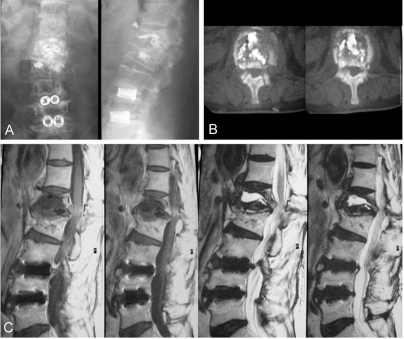
Three months after vertebroplasty, evidence of severe pyogenic spondylitis was seen, including breakdown of the anterior cortex with anterior displacement of the bone cement (**A**, **B**) and a fluid-filled cavitary lesion with a paraspinal abscess like lesion (**C**).

**Fig. 2:**
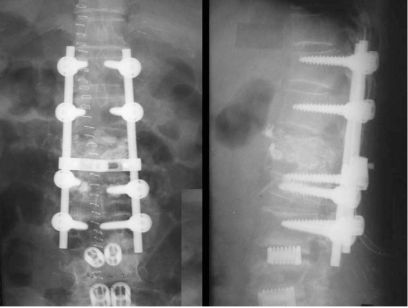
The patient received a pedicle subtraction osteotomy with autogenous bone graft after debridement through a posterior approach.

A 69-year-old female that presented without diabetes and no other immune compromised diseases received vertebroplasty at another hospital for an L1, L2 osteoporotic compression fracture. A postoperative roentgenographic image revealed that bone cement was well localized in the bony trabeculae of the L1 and L2 vertebral body. Three months after surgery, the patient came to our hospital suffering from severe low back pain, mild fever, and progressive weakness of both lower legs. At that time, the body temperature was 37.5℃, the white blood cell (WBC) count was 9,360/µL, the erythrocyte sedimentation rate (ESR) was 28 mm/h, and the level of C-reactive protein (CRP) was 19.1 mg/dL. A roentgenographic image revealed a breakdown of the anterior cortex of the L1 vertebral body, with anterior displacement of the bone cement (Fig. 1A and B). A magnetic resonance image showed a fluid-filled cavitary lesion of the L1 vertebral body and a paraspinal abscess like lesion with invasiveness of the spinal canal (Fig. 1C). After broad antibiotic therapy for 3 weeks, the body temperature was 36.5℃, the WBC count was 10,000/µL, the ESR was 26 mm/h, and the CRP level was 3.5 mg/dL. Transpedicular subtraction osteotomy with a bone graft was performed after debridement (Fig. 2). The severe back pain with associated weakness improved after surgery.

### Case 2

**Fig. 3:**
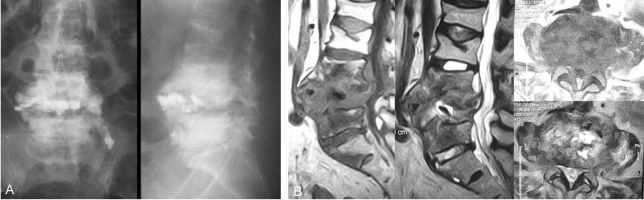
At 34 months after the vertebroplasty (the first visit to our hospital), a roentgenographic image (**A**) and MRI (**B**) revealed a sclerotic change of the vertebral body, with anterior displacement of the bone cement, and multiple paraspinal and intraspinal abscesses with invasion of the spinal canal.

**Fig. 4:**
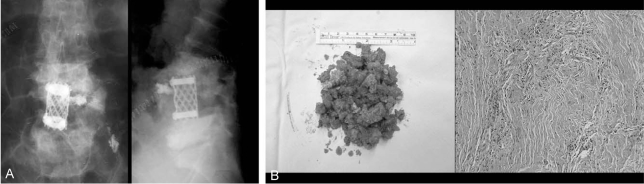
The patient received an anterior corpectomy with a strut mesh cage (**A**) and the pathological report (**B**) showed the presence of osteochondral tissue with degeneration, as well as chronic inflammation and fibrosis.

A 74-year-old female that presented without diabetes and no other immune compromised diseases received a laminectomy with vertebroplasty for L4-5 spinal stenosis and an L4 osteoporotic compression fracture at another hospital. A post operative wound did not heal well and a serous discharge was drained consecutively. To control the infection of the surgical wound, debridement was performed twice. After two months, MRI showed endplate destruction and a cavitary lesion like an abscess at the site of L4. After 6 months and 22 months, serial roentgenographic images revealed a breakdown of the anterior cortex of the L4 vertebral body, with anterior displacement of the bone cement, and a sclerotic change of the L4 and L5 vertebral body. Three years after surgery, the patient came to our hospital and complained of severe back pain radiating to the lower abdomen, with associated weakness of both lower legs and walking difficulty. At that time, the body temperature was 37.7℃, the WBC count was 12,200/µL, the ESR was 32 mm/h, and the CRP level was 23.3 mg/dL. A roentgenographic image and MRI revealed a sclerotic change of the vertebral body, with anterior displacement of the bone cement, and multiple paraspinal and intraspinal abscesses with invasion of the spinal canal (Fig. 3). After broad antibiotic therapy for one week, the body temperature was 36.7℃, the WBC count was 10,900/µL, the ESR was 67 mm/h, and the CRP level was 19.6 mg/dL. Because of the poor general condition of the patient, temporary percutaneously endoscopic irrigation, debridement and a biopsy were performed under local anesthesia. After 3 weeks, a left-side abdominal approach for a total corpectomy of L4-L5 and decompression of the spinal canal were performed. None of the bone cement and infected bone was removed completely as the bone was hard like granite and close to the major neurovascular structures. An autologous iliac bone graft with a titanium mesh cage was performed after debridement (Fig. 4A). The pathology report indicated the presence of osteochondral tissue with degeneration as well as chronic inflammation and fibrosis (Fig. 4B). After 3 months, the patient was eventually discharged after the condition had stabilized.

## Discussion

The current indications for vertebroplasty include compression fractures due to osteoporosis as well as osteolytic metastases and spinal myeloma lesions. With the number of percutaneous vertebroplasties performed steadily increasing, complications have also risen, though a complication is still very rare. Complications include asymptomatic cement leakage, cardiovascular effects, and embolisms with a lethal outcome as well as severe neurological deficits^2^-^4^. Over the last several years, an increasing number of cases with varying complications, their genesis, and their management have been reported in the literature. To the best of our knowledge, three cases of infectious complications requiring surgical management have been reported. Only one case reported in the literature required conservative antibiotic therapy and restriction of movement.

Yu et al.^5^ reported in one case that for a patient under the impression of urinary tract infection, cefazoline and gentamycin antibiotics were adminstered for a week, then vertebroplasty was performed once the patient was no longer febrile. One month later, the patient received an anterior interbobody fusion with a strut bone graft after debridement and posterior instrumentation.

Walker et al.^6^ presented two patients where osteomyelitis developed after vertebroplasty in which a corpectomy was required. Schmid et al.^7^ reported one case where a patient was treated with intravenous ciprofloxacin and consecutive clindamycin for a total of 3 months. A 55-year-old patient with secondary osteoporosis due to liver cirrhosis from alcohol abuse was described, in which a percutaneous vertebroplasty of three fractured vertebral bodies (L3-L5) was complicated by spondylitis. One year after the infection, the MRI signs of spondylitis resolved without further collapse of L5. In the presented case, a satisfying result was achieved through conservative antibiotic therapy and restriction of movement.

In this report, we have presented two patients where osteomyelitis developed after vertebroplasty in which a corpectomy was required. Yu et al.^5^ have reported that the cause of infection is due to the the timing of surgery. It is doubtful that a urinary tract infection could cause systemic bacteremia with a high risk of operative complications, but surgery should not proceed until complete recovery from infection. Furthermore, in a case where a recent infection had been diagnosed, antibiotics should be administered on a long-term basis after surgery to prevent deep infection, or a cement-antibiotics mixture should be used.

We have treated more than 100 cases without using a cement-antibiotic mixture (Depuy vertebroplastic cement), and no infection had been detected. Even though Moreland et al.^8^ thought that the addition of antibiotic powder (Tobramycin; Abbott, Chicago, IL, USA) to PMMA during preparation had not demonstrated a benefit. Of the reports that were reviewed, only one infection was reported in 682 cases, and this was in immune-compromised patients who developed spondylodiscitis. Nevertheless, we recently always mix the antibiotics with the vertebroplastic cement after the unfortunate experience of our cases.

The most effective treatment method for complications concerned with surgery is prevention. Factors increasing the infection rate after surgery are poor nutrition, obesity, diabetes, long-term use of steroids, prolonged admission, an underlying infectious disease and other causes. With consideration of these factors, we can assume by analogy that most patients with osteoporotic compression fracture are highly susceptible to infection. Therefore, we should be mindful of the risks of postoperative infection and keep in mind the benefits of preoperative careful evaluation and attention. Even though infection rates after vertebroplasty are statistically very low, such complications would be disastrous for the patients.

Vertebroplasty should be performed under sterile conditions. Excluding the possibility of spinal infection before surgery and a detailed evaluation, it is crucial that the patient is without systemic infectious disease before the vertebroplasty procedure is performed.
